# A Computational Framework Analysis of Public Attitudes Toward Male Human Papillomavirus Infection and Its Vaccination in China: Based on Weibo Data

**DOI:** 10.3390/healthcare13030287

**Published:** 2025-01-31

**Authors:** Xuan Zhou, Hao Gao, Jun Wang

**Affiliations:** School of Journalism and Communication, Nanjing Normal University, Nanjing 210097, China; 230212033@njnu.edu.cn (X.Z.); 42396@njnu.edu.cn (H.G.)

**Keywords:** male HPV vaccination, Sina Weibo, public attitude, ANTMN

## Abstract

Background/Aims: The global promotion of HPV vaccines has underscored the importance of vaccination for both males and females in reducing disease transmission and associated complications. Despite robust evidence supporting male HPV vaccination, China has yet to approve it. Public discussions on male HPV vaccination, influenced by policy delays, gender norms, and commercialization, reveal diverse attitudes and significant challenges in achieving equitable health outcomes. This study investigates public perceptions and attitudes toward male HPV vaccination in China by analyzing cognitive frames and the social, cultural, and economic factors shaping these discussions. Methods: This study employs a cross-sectional design to analyze 4997 Sina Weibo posts using the Analysis of Topic Model Networks (ANTMN), identifying five frames: Disease Risk and Prevention, Virus Transmission, Gender Roles and Perceptions, Vaccine Promotion and Acceptance, and Market Dynamics and Consumption. Results: The findings reveal a significant gap between policy implementation and public awareness of male HPV vaccination in China. Despite growing recognition of its benefits, entrenched gender norms and biases hinder equitable health outcomes. Social media, as a pivotal platform for health communication, plays a dual role in facilitating public discourse while also amplifying misinformation. Policy delays and concerns over vaccine commercialization heighten public hesitancy, emphasizing the need for inclusive policies and targeted education. Conclusions: This study highlights the necessity for systemic efforts to address gender biases, enhance public education on male HPV vaccination, and rebuild trust in vaccination programs. A multifaceted approach is required to bridge these gaps, integrating policy reforms, inclusive health communication strategies, and equitable vaccine access. These measures aim to promote awareness and increase vaccination uptake among males in China, ultimately contributing to more comprehensive and equitable public health outcomes.

## 1. Introduction

With the global promotion of Human Papillomavirus (HPV) vaccines, the importance of HPV vaccination for males has gradually gained attention [1]. Both males and females are susceptible to HPV infection [2]. Globally, 31% of males aged 15 and above are infected with at least one type of genital HPV, and 21% are infected with at least one high-risk or carcinogenic HPV type [3]. In China, the overall HPV infection rate in the general population is approximately 13.1% to 18.8%, with an HPV infection rate of 14.5% in heterosexual males’ genital and anal regions. The infection rate is even higher among the MSM (men who have sex with men) population, reaching 59.9% [4]. Vaccination is an effective measure for preventing and controlling HPV infections and their complications [5]. Countries like the United States and Australia have included HPV vaccination for males in their national immunization programs to expand cancer prevention coverage [6]. However, HPV vaccination for males has not yet been approved in China [7]. In 2023, Merck submitted an application to the China Center for Drug Evaluation for the male indication of the nine-valent HPV vaccine, which, if approved, will mark significant progress in HPV prevention and control for males in China [8]. Similar to the issue of policy coverage insufficiency, public awareness of male HPV infection and the necessity of vaccination also remains relatively weak, reflecting a dual gap in policy and public understanding of male HPV prevention in China [9].

Media attention to public health issues is typically event-driven [10]. With the trial and promotion of the male HPV vaccine in China, discussions on male HPV infection and vaccination have also sparked widespread debate on Chinese social media. On the one hand, some members of the public support vaccination for males, arguing that it can protect their partners’ health while enhancing their health protection. On the other hand, skepticism has arisen, focusing on the purpose, economic benefits, and necessity of promoting HPV vaccination for males. These disagreements reflect the diversity in public understanding of vaccines and highlight the role of social media in reinforcing the gender labeling and commercialization of HPV vaccines [11]. In social media discussions, HPV vaccines have long been labeled as “feminine” [12], with the public generally associating them with cervical cancer prevention while overlooking the health benefits of male vaccination. Furthermore, negative discussions about the high cost of vaccines, limited supply, and potential side effects have heightened public hesitancy toward vaccination [13]. As a vital channel for health communication, social media plays a dual role in this process, facilitating the dissemination of information while also amplifying public distrust through the rapid spread of negative content [14].

Current research on HPV vaccines primarily focuses on attitudes and acceptance among females, with relatively fewer studies addressing male HPV infection and vaccination [15]. International studies reveal that HPV vaccination rates among males are significantly lower than among females [16], constrained by multiple factors such as lack of knowledge, insufficient medical recommendations, economic costs, and societal gender norms [1]. Additionally, sexual orientation also influences male perceptions and vaccination behaviors related to HPV [17]. Despite the apparent health benefits of HPV vaccination for males and its role in reducing cervical cancer risk in females, these advantages have not significantly increased male interest in the vaccine [17]. In China, research on male HPV vaccination primarily focuses on regional differences in vaccination awareness and willingness, with male college students as the primary study population. Studies have found that knowledge level, sexual education, and economic conditions are crucial factors influencing HPV vaccination among college students [18]. Furthermore, male college students generally exhibit high hesitancy toward the nine-valent HPV vaccine, although those with medical-related academic backgrounds or romantic relationships demonstrate stronger vaccination willingness [19]. While existing studies have preliminarily identified individual and social factors affecting male HPV vaccination, they are mostly limited to specific populations, such as college students, and fail to reflect HPV awareness and vaccination intentions among Chinese males comprehensively. On a broader societal level, cognitive barriers and mechanisms to promote HPV vaccination for males require further exploration.

At the same time, the information dissemination environment, as a critical factor influencing public perceptions, plays a key role in the issue of male HPV vaccination. Traditional media in China often align with government policy developments and pay limited attention to topics related to male vaccination [20]. In contrast, social media provides a more diverse space for discussions on HPV-related topics, serving as a significant source of HPV-related information for the public [12] and exerting a notable impact on public perceptions and attitudes [21]. However, the spread of negative information on social media has also significantly influenced public perceptions and decision-making regarding vaccination [22].

As public discussions gather on online platforms, skepticism and accusations regarding male HPV vaccines have also been widely disseminated on social media [23]. This skepticism stems not only from controversies surrounding the cost and efficacy of the vaccine [24] but also from the influence of gender labeling. From a gender perspective, HPV has long been associated with cervical cancer in women, leading to its “feminization” and reinforcing gender biases while neglecting the cost-effectiveness of vaccination for males [25]. This further limits the potential benefits that males could gain from the HPV vaccine. At the same time, social media has reinforced unequal gender relations and the allocation of health responsibilities in the promotion of HPV vaccines. Women are positioned as the primary targets for vaccination, while the health needs of men are marginalized [11].

Although issues of gender labeling and commercialization of HPV vaccines frequently appear in public discussions, existing research on the interaction mechanisms between gender norms and vaccine perceptions on Chinese social media remains limited. Furthermore, the spread of vaccine conspiracy on social media and their potential impact on public vaccine decisions have not received sufficient attention. Against the backdrop of increasing vaccine commercialization and a diversifying public discourse, public attitudes toward vaccination are influenced not only by health information but also by societal gender norms and skepticism of commercial motives. These factors collectively create a complex social landscape for promoting male HPV vaccination. Based on this, this study focuses on public discussions related to male HPV infection and vaccination on social media, aiming to address the following research questions:

Q1. What cognitive frames regarding male HPV vaccination are presented in social media discussions?

Q2. What do these cognitive frames reveal about the public’s scientific understanding of male HPV vaccination and its promotion?

Q3. From a gender perspective, what biases and inequalities exist in public discussions about male HPV vaccination?

## 2. Materials and Methods

### 2.1. Study Design

This study adopts a cross-sectional research design to explore the perceptions and attitudes of the Chinese public toward male HPV infection and vaccination on social media platforms. Collecting 4997 Weibo posts from a specific period in 2024, this study examines the public’s cognitive framework regarding male HPV vaccination. The cross-sectional design is suitable for capturing public attitudes and discussion trends at a particular time without involving follow-up or long-term observation. Therefore, the data collection reflects the public’s cognitive and emotional responses to the issue at that specific moment.

### 2.2. Materials

This study aims to explore the perceptions and attitudes of the Chinese public toward male HPV infection and vaccination on social media platforms. Sina Weibo was selected as the data source to obtain high-quality data related to male HPV. As one of China’s most popular social media platforms [26], Sina Weibo boasts a large user base with significant penetration among younger demographics. Its information dissemination is characterized by real-time and public interactions [27], making it a suitable platform for capturing public responses to health information and policy updates. Additionally, Weibo’s unique “hashtag” feature facilitates concentrated discussions on specific topics [28], generating substantial textual data to support this study.

This study utilized keywords such as “male HPV” to search for Weibo posts and employed a crawler tool based on the API, supported by the Octoparse platform, for data collection. During the data preprocessing phase, Python scripts were used to remove duplicate content and filter out posts published by official government or media accounts, ensuring the data closely reflected the public’s authentic opinions. To further enhance the relevance and validity of the data, posts unrelated to or loosely connected with the research topic were excluded. Ultimately, the study yielded 4997 valid Weibo posts.

### 2.3. Methods

This study employs the Analysis of Topic Model Networks (ANTMN) and textual analysis to process Weibo posts related to male HPV infection and vaccination. ANTMN, developed by Walter et al., combines topic modeling and semantic networks to identify latent topics and frames in media coverage [29]. This method automatically identifies and constructs topics from data rather than relying on predefined theories or assumptions [30], which helps reduce bias and subjectivity in analysis, making frame analysis more objective and accurate. It also enables the automatic extraction and analysis of information from large volumes of text data, uncovering hidden frame structures [31], thereby providing comprehensive and detailed analytical results.

Moreover, the same vocabulary can appear across multiple topics, avoiding the isolation of individual themes [32]. This flexibility allows frame analysis to reflect the complexity and diversity of the text more accurately. Textual analysis, an essential qualitative research method, delves deeper into hidden messages, thematic patterns, and social meanings within texts. In this study, textual analysis complements the ANTMN by interpreting the visualized quantitative results, summarizing them, and uncovering the core cognitive frames and discussion logic of Weibo users regarding male HPV infection and vaccination. The operational steps are detailed below.

#### 2.3.1. Text Preprocessing

The raw textual data were systematically preprocessed to ensure data quality and analysis validity. The primary goal of text preprocessing was to reduce noise levels and improve the accuracy and efficiency of subsequent analyses.

First, the OpenCC tool was used to convert Traditional Chinese text into Simplified Chinese, addressing potential inconsistencies in character sets within Chinese text data and ensuring uniform text formatting. Next, the Tsinghua University Medical Dictionary was imported and integrated with the Jieba segmentation tool for specialized word segmentation. Jieba utilizes a Chinese word segmentation algorithm based on the Hidden Markov Model (HMM), employing probabilistic statistical methods to predict word boundaries, enabling precise identification of medical terms in the text and enhancing segmentation performance [33].

After segmentation, stopword filtering was conducted using the Baidu Chinese stopword list. Stopwords are high-frequency words that do not contribute significant semantic value in natural language processing, such as common function words [34]. This filtering step helps remove low-information words, improving the semantic focus of the text. Additionally, regular expressions were employed to remove special characters from the text, retaining only Chinese and English characters to ensure data consistency and adherence to analytical standards.

In the vocabulary filtering process, single-character words, often semantically sparse and low in informational contribution, were removed to enhance semantic completeness. Furthermore, specific low-information words were predefined and excluded to mitigate data redundancy’s impact on topic modeling within the context of this study.

#### 2.3.2. Topic Extraction

After completing text preprocessing, this study employed the Latent Dirichlet Allocation (LDA) model to extract topics and uncover latent semantic structures within the text data. LDA is a probabilistic topic model based on Bayesian inference, assuming that each document is composed of multiple latent topics and each topic is generated by a probability distribution over words [35]. Specifically, LDA statistically analyzes the document-term matrix to generate a document-topic and topic-term distribution matrix. The mathematical formula for LDA is as follows:

(1) $$P(w|\theta ,\emptyset )=\prod_{n=1}^{N}\sum_{k=1}^{K}\theta_{d,k}\emptyset_{k,w_{n}}$$

Before training the model, a document-term matrix was constructed, and term weights were adjusted using the Term Frequency–Inverse Document Frequency (TF-IDF) method to enhance the representational ability of significant terms. The TF-IDF formula is as follows:

(2) $$TF\left(t,d\right)=\frac{f_{t,d}}{\sum_{k}f_{k,d}}$$

(3) $$IDF(t)=\log\frac{N}{1+n_{1}}$$

(4) TF−IDF(t,d)=TF(t,d)·IDF(t)

where:

- TF(t,d): Term frequency of word t in document d.
- IDF(t): Document frequency of word t across all documents.
- N: Total number of documents.

The optimal number of topics was determined by adopting an iterative approach, with incremental increases in the number of topics and multiple experiments conducted while observing changes in the perplexity curve. Perplexity, a key metric for evaluating model performance, indicates how well the model fits the data; the lower the perplexity, the better the model’s performance, and the more accurate the topic division [36].

(5) $$Perplexity=exp\left(-\frac{\sum_{d=1}^{D}\log P\left(w_{d}\right)}{\sum_{d=1}^{D}N_{d}}\right)$$

Ultimately, 24 topics were selected as the optimal number for this study. During LDA model training, adaptive hyperparameters (alpha = “auto”; eta = “auto”) were used to ensure smoother topic distributions aligned with the characteristics of the data. Additionally, the model underwent 20 iterations (passes = 20) to enhance its stability and ensure convergence and accuracy of results. For each extracted topic, the top 10 keywords with the highest probabilities were selected as representative terms to characterize the core semantics of the topic.

#### 2.3.3. Topic Network Construction

This study constructed a topic network based on Pointwise Mutual Information (PMI) to explore the in-depth relationships between topics. PMI is a semantic association metric that measures the co-occurrence relationships of terms or topics, reflecting their statistical dependence [37,38].

First, the document-topic distribution matrix (θ) was obtained from the LDA model. This matrix represents the probability distribution of each topic within a document and serves as the foundation for analyzing inter-topic relationships. Based on this distribution, a co-occurrence probability matrix was calculated by measuring the probability of different topics appearing together in the text. Subsequently, the PMI formula was applied to quantify the strength of semantic association between topics. The formula is as follows:

(6) $$PMI\left(x,y\right)=\log_{2}\left(\frac{P\left(x,y\right)}{P\left(x\right)P\left(y\right)}\right)$$

where:

- P(i,j) is the co-occurrence probability of topics i and j.
- P(i) and P(j) are the marginal probabilities of topics i and j, respectively.

A higher PMI value indicates a stronger semantic association between topics i and j; conversely, a PMI value close to zero or negative suggests weak or nonexistent relationships between the topics. A PMI threshold was set to filter out significant associations. Only topic pairs exceeding this threshold were considered strongly associated and included in the topic network. The network was defined as G = (V, E), where V represents the set of topic nodes, and E represents the edges connecting strongly associated topics. Finally, the constructed topic network was exported in GEXF format for further network analysis and visualization. Additionally, the keyword lists for each topic were exported into an Excel file to facilitate qualitative analysis and provide a more explicit interpretation of the specific content of each topic.

#### 2.3.4. Community Detection and Visualization Analysis

After constructing the topic network, community detection methods were employed to analyze the structural features of the network and reveal clustering relationships between topics. The theoretical basis of community detection is the maximization of network modularity, which identifies tightly connected clusters of nodes within the network [39]. The modularity is calculated using the following formula:

(7) $$Q=\frac{1}{2m}\sum_{i,j}\left[A_{ij}-\frac{k_{i}k_{j}}{2m}\right]\delta \left(c_{i},c_{j}\right)$$

where:

- A_ij_: Weight of the edge between nodes i and j.
- k_i_ and k_j_: Degrees of nodes i and j.
- m: Total weight of all edges in the network.
- delta(c_i_, c_j_): Indicator function, 1 if c_i_ = c_j_, and 0 otherwise.

Before community detection, the average weighted degree of the network was calculated, resulting in 126.097, indicating a high connection strength among nodes and strong associations between topics. Subsequently, the Louvain algorithm in Gephi was employed for community detection. The Louvain algorithm is a heuristic community detection method based on modularity maximization, characterized by its efficiency and suitability for large-scale networks. In terms of parameter settings, this study adopted random initialization, considered edge weights, and set the resolution to 1.0 to ensure the accuracy and stability of community partitioning. After community detection, the study visualized the topic network using Gephi and optimized the presentation of the network graph by adjusting parameters. During the visualization process, elements such as node size, color, and edge thickness were used to display the semantic association structure between topics.

#### 2.3.5. Text Analysis

In addition to quantitative analysis and visualization, this study employed text analysis methods to interpret the data. The first step of the text analysis was to combine the topic model results, focusing on high-frequency words and their contextual usage to identify the implicit meanings and logical connections behind the keywords discussed by Weibo users. By reading sample posts and integrating the topic distributions with the community structures in the network graph, this study explored the discussion patterns and framing directions represented by each topic node, ultimately summarizing the main cognitive frames of Weibo users.

The text data were categorized into five core frames based on the results of the quantitative analysis. Each frame represents a distinct perspective and discussion logic regarding male HPV infection and vaccination. The summary and analysis of these frames reveal the primary concerns of Weibo users and clarify the dissemination pathways and impact of public opinion on these issues in society. Additionally, the text analysis emphasized uncovering metaphors and symbols in the context to investigate users’ implicit attitudes and emotional inclinations. This approach provided more profound insights into the diverse perspectives and potential contradictions expressed by Weibo users when discussing male HPV vaccination.

## 3. Results

**Figure 1:**
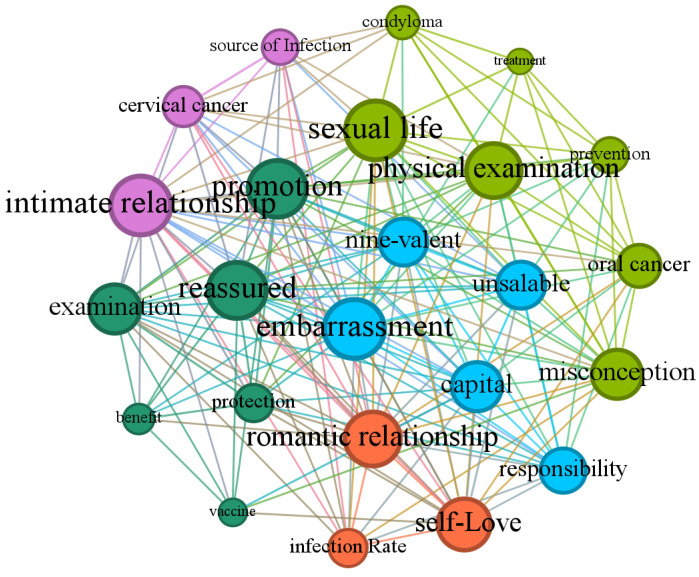
Semantic network.

Based on data from Sina Weibo, this study utilized topic modeling and semantic network analysis to extract and summarize five cognitive frames (Figure 1): “Disease Infection and Transmission”, “Gender Roles and Perceptions”, “Vaccine Promotion and Acceptance”, “Vaccine Market and Consumption”, and “Disease Risk and Prevention”. These frames highlight the focal points of public discussions and reflect the multifaceted influences of scientific understanding, gender culture, and marketization in vaccine promotion.

### 3.1. Disease Risk and Prevention Frame

This frame focuses primarily on the transmission risks and preventive measures related to HPV-associated diseases, as shown by the light green nodes in Figure 1, accounting for 25% of the corpus. Keywords such as “genital warts”, “medical checkup”, “misconceptions”, “treatment”, “prevention”, “oral cancer”, and “sexual activity” reflect public perceptions of HPV-related disease risks, preventive behaviors, and current levels of awareness.

First, this frame emphasizes issues related to male HPV infection from the perspective of risk perception. Although the risks of male HPV infection have been widely publicized, public discussions often concentrate on transmission through sexual behavior, with less attention paid to non-sexual transmission routes. For instance, science communication posts on Weibo frequently mention, “the overall genital HPV infection rate among men is as high as 45.2%”, listing associated disease risks such as “anal cancer”, “genital warts”, and “penile cancer” [40]. These posts provide scientific evidence for the health consequences of male HPV infection. However, some users express misconceptions, such as “If you don’t date, you don’t need the vaccine” [41], highlighting persistent limitations in HPV risk awareness.

Second, this frame underscores the importance of early screening and scientific prevention for male health. Keywords such as “checkup”, “screening”, “medical examination”, “doctor”, and “hospital” appear frequently, indicating public interest in early intervention measures for men. Many users believe that men, like women, should undergo regular HPV tests and screenings, particularly in romantic or marital relationships, where male screening is deemed crucial for the health of both partners. For example, trending topics on Weibo, such as “couples cohabitating should test for HPV” and “the cause of a wife’s HPV recurrence is the husband”, reflect growing public awareness of male HPV prevention.

However, current prevention campaigns primarily focus on cervical cancer screening for women, with insufficient scientific prevention outreach targeted at men. Additionally, many related discussions are intertwined with women’s health measures, reducing the independence of male health issues. In this context, science communication content plays a vital role in improving male awareness of HPV risks. Some posts clarify common misconceptions, such as “HPV is not solely transmitted through sexual contact”, “men also need the HPV vaccine” and “the vaccine is not a cure-all”. Through the dissemination of such content, the public increasingly recognizes that HPV infection is not solely a women’s health issue and that its association with male diseases deserves equal attention. This shift in awareness has spurred further discussions on scientific preventive behaviors for men on Weibo. For example, some users noted that male HPV vaccination is not only an act of responsibility toward their partners but also a means of protecting their health [42].

Finally, this frame highlights women’s expectations for men to adopt scientific preventive behaviors. Female users frequently express support for male participation in HPV prevention and criticize some men for neglecting health risks, reflecting distrust toward male preventive behaviors. For instance, one user commented bluntly, “Men who don’t get vaccinated or tested are irresponsible” [43]. This sentiment underscores increasing public focus on male responsibility in HPV prevention, although improving actual male participation remains a challenge.

### 3.2. Virus Infection and Transmission Frame

The second frame primarily focuses on the sources of HPV infection, transmission routes, affected populations, and the associated health risks of related diseases. The purple nodes in Figure 1 represent this frame for 29.17% of the corpus. Keywords include “infection source”, “cervical cancer”, and “intimate relationships”.

Under this frame, Weibo users demonstrate significant interest in the mechanisms of HPV transmission and its associated health threats, particularly the pervasive and latent nature of the virus. Although many science communication accounts actively disseminate scientific knowledge about HPV infection on the platform, user discussions still reveal limitations. Many users focus on sexual contact as the sole transmission route, commonly perceiving sexual partners as the primary source of infection while overlooking other potential transmission pathways, such as shared hygiene items and mother-to-child transmission.

Additionally, female users are the primary contributors to discussions about male HPV infection and vaccination. In conversations about virus infection and transmission, they frequently mention men and intimate relationships, gradually recognizing the possibility of male HPV infection and tending to view men as potential infection sources. For example, one user commented, “Men carry the virus without symptoms, and women develop symptoms upon contact” [44]. This gendered perspective reinforces the understanding of sexual behavior as the main transmission route, further emphasizing the singular focus of discussions on transmission. Consequently, the public’s neglect of HPV’s complex transmission mechanisms leads to skewed perceptions of disease prevention and control.

Despite the abundance of educational content on Weibo about male HPV infection and related diseases, user discussions often rely on the generalized term “HPV infection” rather than delving into specific diseases. Alternatively, they directly associate HPV infection with “cervical cancer” and sexually transmitted diseases without fully acknowledging its connection to various other conditions. This limited understanding reflects a need for more comprehensive public education on the diverse health risks associated with HPV.

### 3.3. Gender Roles and Perceptions

The third frame focuses on public health perceptions regarding male HPV infection and vaccination, as well as gendered health behaviors. Represented by the orange nodes in Figure 1, this frame accounts for 12.5% of the corpus. Keywords such as “infection rate”, “relationship”, and “self-love” reflect the complexity and contradictions of gender roles in health-related discussions.

First, the role of men as HPV carriers and the societal neglect of male health risks emerged as key discussion topics. Despite research confirming high HPV infection rates among men and its association with various diseases, public perception still assigns women greater responsibility for actively managing health. In contrast, men are often seen as passive participants in the role of “protectors”. In romantic and marital relationships, this “protector” role is often framed as a responsibility toward their partner’s health rather than a commitment to self-protection. For example, one user noted, “If you are in a long-term heterosexual relationship, getting the HPV vaccine is not just about protecting yourself but also about protecting your partner” [45]. This gendered health perspective reveals a lack of male attention and proactiveness regarding their own HPV infection risks.

Second, this frame highlights the tension between health responsibility and gender perceptions. On the one hand, female users on Weibo advocate for male vaccination, emphasizing equal health responsibilities for both genders. They frequently compare the higher uptake of male HPV vaccination in other countries and criticize the perceived lack of male responsibility in HPV prevention in China. On the other hand, traditional moral narratives still dominate, linking the prevention of HPV infection with moral discipline, particularly for women, by imposing stricter behavioral standards. For instance, some users argue that women can avoid infection risks through “self-love” or “purity” [46].

However, this moralistic narrative overlooks the importance of scientific knowledge. It shifts the focus of sexually transmitted disease prevention to women’s moral character while further diminishing the active role of men in health management. Such gendered storytelling not only hinders the dissemination of scientific understanding but also creates significant gaps in gender equality regarding HPV prevention and vaccination.

### 3.4. Vaccine Promotion and Acceptance Frame

The fourth frame focuses on public discussions surrounding the promotion strategies and acceptance of HPV vaccines. The green nodes in Figure 1 represent this frame for 12.5% of the corpus. Keywords such as “protection”, “checkup”, “promotion”, “vaccination”, “vaccine”, and “benefit” highlight the promotional models of vaccines in various social contexts, users’ attitudes toward vaccination, and the level of acceptance.

This frame first underscores the role of social media as a key platform for promoting HPV vaccines. Weibo users utilize hashtags to participate in topic discussions and share information, thereby advancing public knowledge of male HPV infection and vaccination. Science communication accounts and medical professionals are the primary disseminators of information about male HPV infection and vaccination, providing scientific evidence through professional interpretations. For instance, a post by the Weibo user “Obstetrician Ma Liangkun” emphasized that the HPV vaccine is not exclusive to women and explained the relationship between HPV infection and cervical cancer [47]. Additionally, ordinary users share personal experiences to enhance public awareness of male HPV vaccines. Such posts often highlight the dual benefits of male vaccination for personal and partner health, calling for broader acceptance of male HPV vaccines in China.

With the increasing dissemination of scientific information, some male users have begun expressing their willingness to accept the HPV vaccine and publicly stated their intention to get vaccinated. However, despite the overall upward trend in public acceptance of male HPV vaccines, concerns and resistance persist. On the one hand, as HPV vaccines for men have not been fully approved in China, the public has a limited understanding of the vaccine’s efficacy, potential side effects, and administration process, leading some users to question its safety and necessity. On the other hand, factors such as high vaccine costs, limited availability, and the association of vaccines with sexual behavior act as significant barriers to male vaccination willingness.

Some men perceive vaccination as “embarrassing” or “awkward”, diminishing their willingness to get vaccinated. For example, news about South Korean singer Cho Kyuhyun receiving the HPV vaccine sparked controversy, with some users associating male vaccination with “improper sexual behavior”. They questioned his private life and claimed that “only promiscuous men need the nine-valent vaccine” [48]. Such societal and cultural biases reinforce the notion that ″as long as you stay pure and don’t mess around, there’s no need for the vaccine″ [49]. These stereotypes perpetuate misconceptions and hinder the normalization of male HPV vaccination.

### 3.5. Vaccine Market and Consumption Frame

The fifth frame focuses on the current consumption patterns of HPV vaccines in the market, gendered marketing strategies, and public attitudes toward the operation of the vaccine market. The blue nodes in Figure 1 represent this frame for 20.83% of the corpus. Keywords such as “nine-valent”, “not selling”, “capital”, “responsibility”, and “awkwardness” reflect the significant marketization of vaccines in promotion and consumption while also revealing issues of gender division and social equity in the market.

First, discussions in this frame highlight the gendered consumption model of HPV vaccines. Currently, the HPV vaccine market in China is primarily targeted at women, and vaccines for men have yet to be approved, further reinforcing gender divisions within the vaccine market. Many Weibo users criticized the “female-only” positioning of the vaccine market in China, arguing that this approach obscures HPV as a universal health threat. For example, one user commented, “This domestic practice of only letting women get the HPV vaccine makes the public think only women are affected, blurring the real cause”, calling for the opening of the male vaccine market to achieve gender equality in health protection [50].

Second, vaccine pricing and the supply-demand imbalance emerged as focal points of public discussion within this frame. Keywords such as “not selling” and “capital” frequently appeared, reflecting dissatisfaction with high vaccine prices and concerns about capital-driven operations. On the one hand, the nine-valent vaccine is in short supply, leading to difficulties in booking appointments and high costs, which some individuals, especially men, find unaffordable. On the other hand, high prices are perceived as a result of profit-driven motives. Many users expressed concerns about the “capitalization” of the vaccine market, believing that high pricing diverges from the original intent of public health protection and has turned into a tool for commercial profit.

For instance, discussions about introducing Gardasil, which is suitable for male vaccination in China, generated significant attention on Weibo. However, many users expressed distrust toward Merck [51], accusing the company of “marketing” the male vaccine market [52], stating, “They couldn’t sell it to women, so now they’re targeting men” [53]. Some users even described vaccine promotion as “exploitation” [54].

Amid the high prices and unequal resource distribution challenges, some public members actively called for government measures to reduce vaccine costs to ensure broader coverage. Comments such as “Given the average income in China, the price is still too high” and “It’s free abroad but costs extra here; hopefully, it will become free step by step domestically” reflect public expectations for more equitable vaccine access.

Furthermore, social perceptions significantly influence men’s willingness to consume HPV vaccines. Some men feel “awkward” or “uncomfortable” about vaccination, believing the behavior might be linked to “improper sexual conduct”, which affects social acceptance. These cultural biases exacerbate the psychological burden on men in vaccine consumption, indicating that current male HPV vaccine consumption in China is constrained not only by cost and accessibility but also by societal attitudes.

## 4. Discussion

### 4.1. Gendered Health Perceptions and Responsibility Imbalance: The Challenges and Shifts in Public Perceptions of Male HPV Vaccination

Research findings indicate that public perceptions of male HPV infection and vaccination in China exhibit notable gender differences, reflecting an imbalance in the distribution of health responsibilities. First, the public primarily associates HPV with women, with limited recognition of male-related disease transmission and prevention. On Chinese social media platforms, HPV is often directly linked to female-specific conditions such as cervical cancer. At the same time, the severity of male HPV infection and the importance of male vaccination remain overlooked [55]. Women are thus seen as the primary bearers of health responsibility, reinforcing the “feminized” label of HPV vaccination. Second, although public awareness of male HPV infection risks and the significance of scientific prevention has grown through limited health communication efforts, male vaccination is still widely perceived as an adjunct action to “protect their partners” rather than as a necessary measure for safeguarding men’s health. This gendered division of roles and perspectives not only impedes the improvement of male HPV vaccination uptake but also exacerbates the gender imbalance in health responsibilities.

These perceptions are influenced by both national policies and traditional gender norms in China. On the one hand, China’s HPV vaccination program was initiated later and progressed more slowly compared to other developed countries, and male HPV vaccination remains unavailable [13]. Policy advocacy and scientific education on male vaccination remain insufficient. On the other hand, traditional Chinese gender norms expect women to take on moral obligations as “virtuous wives and good mothers” who actively manage family health. In contrast, men are expected to focus on economic responsibilities, play a secondary role, or be absent in health management [56].

However, it is worth noting that public perceptions regarding the necessity of male vaccination and male health autonomy are gradually improving, primarily driven by information dissemination and interaction on social media. Initially, science communication and online discussions on social media have started to highlight the links between HPV and male health, including risks of diseases such as oral cancer and genital cancers. The public is beginning to recognize that HPV is not solely a women’s issue; men are also susceptible to infection and can act as potential carriers of the virus. This scientific communication has effectively broadened the public’s understanding of HPV, reducing the marginalization of male health concerns. Furthermore, women on social media have played a pivotal role in challenging the unequal distribution of gendered health responsibilities and advocating for shared health management between genders. Discussions about HPV infection and vaccination on Chinese social media are predominantly led by female voices [57]. As societal perceptions evolve and science communication deepens, women are increasingly recognizing the inequities in health responsibility distribution related to HPV and actively calling for men to take on greater responsibilities in vaccination and disease prevention.

### 4.2. Social Media and Health Education Dual Constraints: The Phenomenon and Causes of Singularized HPV Cognition

In discussions of HPV infection on Chinese social media, the problem of singularized public cognition is particularly prominent. On the one hand, public discourse overly focuses on sexual transmission, simplifying HPV infection as being exclusively linked to sexual behavior while paying insufficient attention to other potential transmission routes. On the other hand, while the risks of male HPV infection are highlighted on social media, public recognition of the feasibility and necessity of male HPV vaccination remains incomplete. This singularized cognition limits the public’s comprehensive understanding of HPV transmission mechanisms and reinforces the stigmatization and moral judgment of both infections and vaccines, further exacerbating social prejudice.

Research indicates that the limitations of Chinese social media’s communication mechanisms and the systemic deficiencies in health education are key contributors to this singularized public cognition. As an initial point, as an essential platform for health communication, social media platforms such as Weibo serve as primary sources of health information for the general public [58]. These platforms exhibit significant characteristics of empowerment and communication efficiency [59], but their communication models also have inherent limitations. On the one hand, social media platforms provide ordinary users (predominantly female users) with opportunities to voice their opinions, allowing them to discuss, promote, and even critique HPV infections, which reflects an empowering feature. However, due to gender controversies and societal perceptions, public discussions in China tend to amplify the contentious topic of sexual transmission while neglecting other routes of transmission. On the other hand, the fragmented nature and simplified narrative patterns of social media often lead to the “superficiality” dilemma in information dissemination. It makes it difficult for health information to be comprehensively conveyed, further weakening the depth and breadth of scientific knowledge dissemination.

Furthermore, systemic deficiencies in China’s health education efforts regarding HPV prevention have long been apparent [60]. In particular, there is a notable lack of focus on male disease prevention and vaccination advocacy. Traditional health education often focuses on sex education and women, emphasizing the prevention of cervical cancer, but rarely addresses the risks and roles of men in HPV prevention and control, as well as other transmission routes [55]. This gendered health education model further marginalizes the health needs and participation of male populations, weakening their roles in prevention efforts. Consequently, public recognition of male vaccination remains constrained. Although social media is a supplementary channel for health communication and possesses certain advantages in science education, its communication models cannot compensate for the systemic gaps in health education.

### 4.3. The Controversy over Male HPV Vaccines: Policy Delays and Commercial Trust Crisis

Public attitudes toward male HPV vaccines exhibit apparent contradictions: there is both a call for implementing male vaccination policies and concerns over potential safety issues and the commercialization of vaccines.

Some members of the public, particularly women, express expectations for male HPV vaccination on social media. They believe that men should participate in HPV prevention efforts as actively as women, if not more so, to reduce the risk of disease transmission. This phenomenon not only reflects a shift in public health perceptions but also serves as a response to the current vaccination policies in China. With the widespread dissemination of health information, the public gradually recognizes the scientific rationale and importance of male HPV vaccination. Additionally, compared to countries where HPV vaccines are universally covered, the supply–demand imbalance of the nine-valent HPV vaccine in China is particularly pronounced [61]. Male vaccination remains unapproved, and such policy delays have prompted public reflection on the gendered nature of vaccination strategies, leading some individuals to question the existing vaccination mechanism.

Meanwhile, the commercialization of HPV vaccines has become increasingly apparent, drawing widespread public criticism regarding underlying capitalist motives and safety concerns. On one hand, many question the motives behind the promotion of male HPV vaccines, perceiving the market expansion as driven primarily by profit and a “re-marketing” strategy following the saturation of the female market. On the other hand, concerns over vaccine safety persist, with the perception that these vaccines are more a product of profit-seeking than a response to genuine public health needs. This impression further exacerbates public doubts about vaccine quality [62].

Vaccine pricing, marketing strategies, and negative incidents significantly impact public perceptions of male HPV vaccination. First, while HPV vaccines are universally provided free of charge in some countries, they have yet to be included in China’s national immunization program. The high price of these vaccines exceeds the affordability of low-income groups [63], which fuels public dissatisfaction and a sense of being exploited. Second, media framing messages instilling fear and vulnerability can promote a greater public acceptance of vaccination [64]. Some vaccine manufacturers utilize trending searches, advertisements, and other marketing methods to emphasize the severity of male HPV infection risks, attempting to tap into broader market opportunities. However, excessive marketing often provokes public resentment and fails to establish societal recognition of male vaccination effectively [65]. Finally, negative incidents related to vaccination may lead to public resistance and distrust [63]. HPV vaccines have been repeatedly linked to safety concerns, reinforcing negative perceptions and vaccine hesitancy [66]. As a result, the trust crisis stemming from the commercialization of HPV vaccines not only undermines the promotion of male vaccination but also weakens public support for vaccine policies.

### 4.4. Implications and Limitations

The findings of this study offer significant clinical implications. This study highlights a gap in public awareness regarding male HPV vaccination in China, particularly the misalignment between policy implementation and public understanding. Although awareness of the benefits of male HPV vaccination is gradually increasing, deeply rooted gender roles and biases continue to act as significant barriers to vaccination uptake. This suggests that healthcare professionals, particularly those in clinical settings, should actively engage in patient education, offering evidence-based information to address misconceptions and promote the benefits of male HPV vaccination. Moreover, concerns about policy delays and vaccine commercialization contribute to public hesitancy, underscoring the need for healthcare providers to advocate for timely and inclusive policies while strengthening patient-provider communication to increase vaccination rates.

From a research perspective, the study’s methodological rigor, such as removing duplicate and irrelevant Weibo posts, ensures the data reflects genuine public opinions rather than narratives shaped by official sources or media outlets. ANTMN (Analysis of Topic Model Networks) provides a robust and scientifically sound framework for analyzing public attitudes toward male HPV infection and vaccination. By identifying key cognitive frameworks, this study delivers a structured understanding of the multifaceted factors influencing public perceptions, underscoring the necessity for further research into how cultural and social factors shape attitudes toward health interventions such as vaccination.

However, this study has several limitations that must be acknowledged. First, as thi study relies exclusively on Weibo data, the findings may not fully represent the entire population, thus limiting the generalizability of the results to broader demographic groups. Second, while the study captures public opinions during a specific period, it does not allow for tracking attitude changes or assessing the long-term impact of health interventions like vaccination campaigns. Additionally, despite efforts to filter out irrelevant content, the analysis of social media data may still be subject to biases arising from how users express themselves and the types of discussions dominant on the platform.

Future research could address these limitations by incorporating a more representative dataset, including posts from diverse social media platforms. Employing longitudinal designs would also enable observing shifts in public attitudes over time and provide a deeper understanding of the sustained impacts of health communication and intervention campaigns.

## 5. Conclusions

This study highlights significant gaps in public awareness, policy implementation, and societal attitudes toward male HPV vaccination in China. Social media discussions reveal challenges, including limited recognition of male health risks and entrenched gender biases that marginalize male health needs. While platforms like Weibo disseminate vital information, they also amplify misinformation and public distrust, hindering vaccine acceptance. Addressing these challenges requires inclusive health policies, public education campaigns, and equitable vaccine access. Efforts should focus on dismantling gender stereotypes, enhancing understanding of male health risks, and rebuilding trust in vaccination programs to foster equitable healthcare outcomes.

## Data Availability

The data presented in this study are available on request.

## References

[1] Grandahl M., Nevéus T. (2021). Barriers towards HPV vaccinations for boys and young men: A narrative review. Viruses.

[2] Lacey C.J., Lowndes C.M., Shah K.V. (2006). Burden and management of non-cancerous HPV-related conditions: HPV-6/11 disease. Vaccine.

[3] Bruni L., Albero G., Rowley J., Alemany L., Arbyn M., Giuliano A.R., Markowitz L.E., Broutet N., Taylor M. (2023). Global and regional estimates of genital human papillomavirus prevalence among men: A systematic review and meta-analysis. Lancet Glob. Health.

[4] The Epidemiological Trend of Human Papillomavirus (HPV) Infection. https://vaxlab.dukekunshan.edu.cn/evidence-db-media/hpv-vax-media/human-papillomavirus-hpv-infection-prevalence-trends/?utm_source=chatgpt.com#_ftn1.

[5] Lu P.-J., Yankey D., Jeyarajah J., O’Halloran A., Elam-Evans L.D., Smith P.J., Stokley S., Singleton J.A., Dunne E.F. (2015). HPV vaccination coverage of male adolescents in the United States. Pediatrics.

[6] Stanley M. (2014). HPV vaccination in boys and men. Hum. Vaccines Immunother..

[7] Lv H., Chen J., Liu J., Lu H. (2023). Knowledge levels and vaccination willingness of HPV among HIV-infected men. Chin. J. AIDS STD.

[8] The HPV Infection Rate is Higher in Men Than in Women! How Much Longer Do Chinese Men Have to Wait to Get the HPV Vaccine?. https://www.thepaper.cn/newsDetail_forward_27605644.

[9] Ran H., Chen Y., Gao J., Guo H., Peng S. (2022). Low awareness of HPV infection and willingness of HPV vaccination among Chinese male college students in the east of China. Front. Public Health.

[10] Shih T.J., Wijaya R., Brossard D. (2008). Media coverage of public health epidemics: Linking framing and issue attention cycle toward an integrated theory of print news coverage of epidemics. Mass Commun. Soc..

[11] Pisciotta M.K. (2012). Gendering Gardasil: Framing Gender and Sexuality in Media Representations of the HPV Vaccine. Master’s Thesis.

[12] Li Y., Guo Y., Lin H. (2022). Feminized virus: A content analysis of social media’s representation of HPV vaccine. Soc. Media Soc..

[13] Wang H., Jiang Y., Wang Q., Lai Y., Holloway A. (2023). The status and challenges of HPV vaccine programme in China: An exploration of the related policy obstacles. BMJ Glob. Health.

[14] Chang C. (2013). Men’s and women’s responses to two-sided health news coverage: A moderated mediation model. J. Health Commun..

[15] Liddon N., Hood J., Wynn B.A., Markowitz L.E. (2010). Acceptability of human papillomavirus vaccine for males: A review of the literature. J. Adolesc. Health.

[16] Shin H., Jeon S., Cho I., Park H. (2022). Factors affecting human papillomavirus vaccination in men: Systematic review. JMIR Public Health Surveill..

[17] Gilbert P., Brewer N.T., Reiter P.L., Ng T.W., Smith J.S. (2011). HPV vaccine acceptability in heterosexual, gay, and bisexual men. Am. J. Men’s Health.

[18] Dai Z., Si M., Su X., Wang W., Zhang X., Gu X., Ma L., Li J., Zhang S., Ren Z. (2022). Willingness to human papillomavirus (HPV) vaccination and influencing factors among male and female university students in China. J. Med. Virol..

[19] Jia S., Pan B., Hong D., Zhang Q., Jiang H., Hong Y., Hong J. (2023). A survey of potential acceptance of 9-valent HPV vaccine among Chinese male college students. Hum. Vaccines Immunother..

[20] Hassid J. (2020). Censorship, the Media, and the Market in China. J. Chin. Political Sci..

[21] Chen L., Ling Q., Cao T., Han K. (2020). Mislabeled, fragmented, and conspiracy-driven: A content analysis of the social media discourse about the HPV vaccine in China. Asian J. Commun..

[22] Margolis M.A., Brewer N.T., Shah P.D., Calo W.A., Gilkey M.B. (2019). Stories about HPV vaccine in social media, traditional media, and conversations. Prev. Med..

[23] Briones R., Nan X., Madden K., Waks L. (2012). When vaccines go viral: An analysis of HPV vaccine coverage on YouTube. Health Commun..

[24] Gerend M.A., Shepherd M.A., Shepherd J.E. (2013). The multidimensional nature of perceived barriers: Global versus practical barriers to HPV vaccination. Health Psychol..

[25] Daley E.M., Vamos C.A., Zimet G.D., Rosberger Z., Thompson E.L., Merrell L. (2016). The feminization of HPV: Reversing gender biases in US human papillomavirus vaccine policy. Am. J. Public Health.

[26] Han E.L. (2018). Weibo and the making of Chinese networked publics: Witness, debates and expertise. Commun. Public.

[27] Li J., Xu Q., Cuomo R., Purushothaman V., Mackey T. (2020). Data mining and content analysis of the Chinese social media platform Weibo during the early COVID-19 outbreak: Retrospective observational infoveillance study. JMIR Public Health Surveill..

[28] Guo L., Wang W., Cheng S., Que X. (2014). Event-based user classification in Weibo media. Sci. World J..

[29] Walter D., Ophir Y. (2019). News frame analysis: An inductive mixed-method computational approach. Commun. Methods Meas..

[30] Akcakir G., Jiang Y., Luo J., Noh S. (2023). Validating a mixed-method approach for multilingual news framing analysis: A case study of covid-19. Comput. Commun. Res..

[31] Walter D., Ophir Y. (2021). Strategy framing in news coverage and electoral success: An analysis of topic model networks approach. Political Commun..

[32] Ophir Y., Walter D., Arnon D., Lokmanoglu A., Tizzoni M., Carota J., D’Antiga L., Nicastro E. (2021). The framing of COVID-19 in Italian media and its relationship with community mobility: A mixed-method approach. J. Health Commun..

[33] Zhang H.-P., Liu Q., Cheng X., Zhang H., Yu H.-K. Chinese lexical analysis using hierarchical hidden Markov model. Proceedings of the Second SIGHAN Workshop on Chinese Language Processing.

[34] Sarica S., Luo J. (2021). Stopwords in technical language processing. PLoS ONE.

[35] Blei D.M., Ng A.Y., Jordan M.I. (2003). Latent Dirichlet allocation. J. Mach. Learn. Res..

[36] Zhao W., Chen J.J., Perkins R., Liu Z., Ge W., Ding Y., Zou W. (2015). A heuristic approach to determine an appropriate number of topics in topic modeling. Proceedings of the BMC Bioinformatics.

[37] Church K., Hanks P. (1990). Word association norms, mutual information, and lexicography. Comput. Linguist..

[38] Bouma G. (2009). Normalized (pointwise) mutual information in collocation extraction. Proc. GSCL.

[39] Fortunato S. (2010). Community detection in graphs. Phys. Rep..

[40] HPV Vaccine in Macau. https://weibo.com/1998179337/M0JEczEZm?refer_flag=1001030103_.

[41] HPV Vaccine Sales Are Sluggish. https://weibo.com/6270241922/OwGY2deeA?refer_flag=1001030103_.

[42] How Long Will Chinese Men Have to Wait for the HPV Vaccine?. https://weibo.com/2420394475/Ohiw6pwkj?refer_flag=1001030103_.

[43] Couples Living Together Should Test for HPV. https://weibo.com/6658352378/Odc59cuxH?refer_flag=1001030103_.

[44] Couples Living Together Should Test for HPV. https://weibo.com/7581648327/OdcnOoxou?refer_flag=1001030103_.

[45] Is It Necessary for Men to Get the HPV Vaccine?. https://weibo.com/1998179337/Mgb8hlRg3?refer_flag=1001030103_.

[46] HPV Vaccine Sales Are Sluggish. https://weibo.com/7602977495/OwK5kju9r?refer_flag=1001030103_.

[47] HPV Vaccine for Men Is Coming Soon. https://weibo.com/6696223001/Nij2ferI2?refer_flag=1001030103_.

[48] Cho Kyu-Hyun Got the Nine-Valent HPV Vaccine. https://weibo.com/1998179337/NdVDsCu4A?refer_flag=1001030103_.

[49] HPV Vaccine Price Has Dropped to Just Over 20 Yuan per Dose. https://weibo.com/7051157562/OzFBq8KLP?refer_flag=1001030103_.

[50] Early Marriage and Childbearing May Trigger Cervical Cancer. https://weibo.com/3703081551/Lak32imdW?refer_flag=1001030103_.

[51] Male Genital HPV Infection Rate Reaches as High as 45.2%. https://weibo.com/5666666438/NimGciV7I?refer_flag=1001030103_.

[52] Male Genital HPV Infection Rate Reaches as High as 45.2%. https://weibo.com/6378490445/NilMB4bGe?refer_flag=1001030103_.

[53] HPV Vaccine Sales Are Sluggish. https://weibo.com/6446490150/OwF4H95Ce?refer_flag=1001030103_.

[54] HPV Vaccine Sales Are Sluggish. https://weibo.com/1176120640/P2uO1qH0m?refer_flag=1001030103_.

[55] Xu Y., Bi W., Liu T., Jiang Y., Wang Q., Fan R. (2021). Factors associated with intention of human papillomavirus vaccination among Chinese college students: Implications for health promotion. Hum. Vaccines Immunother..

[56] Jiang F.S., Liu A.Y. (2024). Co-shaping of Division of Labor Practices and Conceptual Interaction: A Situational Perspective on Chinese Couples’ Marital Satisfaction. Women’s Stud. J..

[57] How Many Prejudices Must Be Overcome to Successfully Receive the HPV Vaccine?. https://www.sohu.com/a/514461675_120094093.

[58] Koskan A., Cantley A., Li R., Silvestro K., Helitzer D. (2022). College students’ digital media preferences for future HPV vaccine campaigns. J. Cancer Educ..

[59] Chen L., Wu X., Li M. (2018). Formation and fragmentation within a networked public sphere: Social media debates on Traditional Chinese Medicine. Telemat. Inform..

[60] Zhang X., Liu C.-R., Wang Z.-Z., Ren Z.-F., Feng X.-X., Ma W., Gao X.-H., Zhang R., Brown M.D., Qiao Y.-L. (2020). Effect of a school-based educational intervention on HPV and HPV vaccine knowledge and willingness to be vaccinated among Chinese adolescents: A multi-center intervention follow-up study. Vaccine.

[61] China V., The HPV Vaccination Rate Is Lower Than Rwandan in China Vaccine China 2018. http://www.cnvax.com/t/10525.

[62] Adhikari B., Cheah P.Y., von Seidlein L. (2022). Trust is the common denominator for COVID-19 vaccine acceptance: A literature review. Vaccine X.

[63] Wong L.P., Han L., Li H., Zhao J., Zhao Q., Zimet G.D. (2019). Current issues facing the introduction of human papillomavirus vaccine in China and future prospects. Hum. Vaccines Immunother..

[64] Yousaf M., Raza S.H., Mahmood N., Core R., Zaman U., Malik A. (2022). Immunity debt or vaccination crisis? A multi-method evidence on vaccine acceptance and media framing for emerging COVID-19 variants. Vaccine.

[65] Wynia M.K. (2007). Public health, public trust and lobbying. Am. J. Bioeth..

[66] Bonanni P., Zanella B., Santomauro F., Lorini C., Bechini A., Boccalini S. (2018). Safety and perception: What are the greatest enemies of HPV vaccination programmes?. Vaccine.

